# Anatomical 3D Modeling Using IR Sensors and Radiometric Processing Based on Structure from Motion: Towards a Tool for the Diabetic Foot Diagnosis

**DOI:** 10.3390/s21113918

**Published:** 2021-06-06

**Authors:** Rafael Bayareh Mancilla, Bình Phan Tấn, Christian Daul, Josefina Gutiérrez Martínez, Lorenzo Leija Salas, Didier Wolf, Arturo Vera Hernández

**Affiliations:** 1 Departamento de Ingeniería Eléctrica/Sección de Bioelectrónica, Centro de Investigación y de Estudios Avanzados del IPN, Av. Instituto Politécnico Nacional 2508, Col. San Pedro Zacatenco, Gustavo A. Madero, Ciudad de México 07360, Mexico; lleija@cinvestav.mx (L.L.S.); arvera@cinvestav.mx (A.V.H.); 2 Centre de Recherche en Automatique de Nancy (CRAN)/CNRS, Université de Lorraine, 2 Avenue de la Forêt de Haye, 54516 Vandœuvre-Lès-Nancy, Lorraine, France; Phan_T@ukw.de (B.P.T.); Christian.Daul@univ-lorraine.fr (C.D.); didier.wolf@univ-lorraine.fr (D.W.); 3 División de Ingeniería Biomédica, Instituto Nacional de Rehabilitación “Luis Guillermo Ibarra Ibarrra” (INR-LGII), Calzada México-Xochimilco 289, Coapa, Ciudad de México 14389, Mexico; josefina_gutierrez@hotmail.com

**Keywords:** diabetic foot, thermal 3D surface, IR radiometric processing, Structure from Motion, medical thermography, infrared sensors, developing world diagnostics

## Abstract

Medical infrared thermography has proven to be a complementary procedure to physiological disorders, such as the diabetic foot. However, the technique remains essentially based on 2D images that display partial anatomy. In this context, a 3D thermal model provides improved visualization and faster inspection. This paper presents a 3D reconstruction method associated with temperature information. The proposed solution is based on a Structure from Motion and Multi-view Stereo approach, exploiting a set of multimodal merged images. The infrared images were obtained by automatically processing the radiometric data to remove thermal interferences, segment the RoI, enhance false-color contrast, and for multimodal co-registration under a controlled environment and a ∆T < 2.6% between the RoI and thermal interferences. The geometric verification accuracy was 77% ± 2%. Moreover, a normalized error was adjusted per sample based on a linear model to compensate for the curvature emissivity (error ≈ 10% near to 90°). The 3D models were displayed with temperature information and interaction controls to observe any point of view. The temperature sidebar values were assigned with information retrieved only from the RoI. The results have proven the feasibility of the 3D multimodal construction to be used as a promising tool in the diagnosis of diabetic foot.

## 1. Introduction

According to the International Diabetes Federation, 463 million people were diagnosed with diabetes mellitus (DM) in 2019. Among them, from 40% to 60% have peripheral neuropathy, as a result of diabetic foot complications [1,2]. Commonly, diabetic foot carries a risk for DM patients since it could lead to amputation below the knee joint as a preventive procedure. Furthermore, DM is responsible for a lower limb amputation every 30 s [3]. Several efforts have been focused on the early detection of the diabetic foot by exploiting medical imaging modalities, such as MRI, radiography, and thermography associated with image processing techniques [4,5]. However, the anticipated diagnosis usually received limited attention in terms of research [6]. Table 1 summarizes the state-of-the-art related to the diagnosis of the diabetic foot with different medical imaging modalities.

Although the study of DM has several approaches in terms of medical imaging, infrared thermography (IRT) has the advantage of being a contactless, non-invasive, and passive method. IRT is an alternative method for monitoring superficial body temperatures to detect diseases since abnormal body temperature is a natural indicator of illness or complications [11,12,13,14]. This technique measures radiometric information to obtain correlations between the skin surface temperature and the underlying physiological process, which is commonly visible as a false-color image [15,16,17]. The IR radiation emitted by the skin surface is detected by an array of microbolometers and interpreted as temperature [17].

The measurement protocols in medical thermography consider external factors such as airflows, room temperature, and humidity. However, the acceptance of the IRT by the medical community still remains a challenge due to the uncontrolled acquisition conditions. Improved standardization and a better understanding of the relationship between temperature and physiology remain in the research stage [18]. Nevertheless, IRT is a continuously evolving technology; in recent years, the development of IR equipment was significant in terms of data acquisition and image processing techniques. The improvements are focused on the automatic analysis of the temperature in the region of interest (RoI) to detect thermal patterns [19,20,21]. Currently, several types of thermal cameras have dedicated software for the digital processing of the data and images produced by the arrays of sensors [22]. The gradual IR medical application has increased, not only due to technological advances but also because IRT does not require the use of ionizing radiation and is a non-invasive procedure. Thus, the patient will never be harmed, and the technique helps to apply a fast examination. IRT has proven to be a valuable imaging technology in terms of clinical quantitative evaluation of pathologies and detection of thermal patterns in the human body [23].

On the other hand, the combination between IR and visible-light modalities is relevant for quantitative studies that can facilitate advanced processing, such as 3D reconstruction, which is particularly essential in the biomedical field. An effective image fusion can allow to retrieve information from the original data and integrates it onto the merged images without any artifacts that would compromise the accuracy. Table 2 summarizes some recent biomedical applications and methods for IR and visible-light fusion.

Although the studies presented in Table 2 apply robust techniques for multimodal image registration, the segmentation is carried out manually for IR images.

Since the IRT procedure has become a valuable tool for the early detection of several diseases, it remains essentially a 2D technique that does not provide useful anatomical information since it only displays partial information of the lower limb [26]. Furthermore, typical 2D thermal images are not optimal in terms of reproducibility. The correlation between thermograms or quantitative analysis of the same object depend on the distance and angle between the sensor and the body under observation. In such a context, a 3D anatomical model could improve clinical pre-diagnosis by providing a more comprehensive and faster inspection of the plantar, lateral, and dorsal regions as a single entity. A 3D model allows visualizing lower limbs in a more exploitable way than the planar information seen in 2D images, since 3D information is useful for studying temperature differences from any viewpoint of the foot surface. Table 3 resumes an overview of the state-of-the-art contributions regarding the 3D reconstruction methods using IR images in the biomedical field.

As IRT implementation advances in the medical community, technological improvements in optical sensors from thermographic cameras and image processing have made the Structure from Motion (SfM) and Multi-view Stereo (MVS) techniques effective and common methods for the estimation of 3D surfaces. These techniques were conceived to have visible-light images as input since they require invariant scale feature detection and matching of homologous 2D structure seen from different viewpoints. Thermal images can be determined using the radiometric information acquired with thermal cameras; however, the structure and texture features are usually not detectable in false-color images. Thus, the fusion of IR and visible-light images seen from identical viewpoints is required when SfM and MVS are applied in SfM algorithms.

The SfM method estimates the three-dimensional shape of a scene using only its two-dimensional projections taken from different viewpoints. The first step lies in the determination of homologous feature points for different images. One of the most common feature point extraction and description methods is the scale-invariant feature transform (SIFT) [31]. The feature vectors (or descriptors) are used to track feature points (most often located on the corner) along the image sequence to find correspondences (groups of homologous points correspond to the same 3D point). A geometric verification is carried out to validate potentially overlapping images using homography [32] as a model in the Random Sample Consensus (RANSAC) algorithm [33]. The SfM algorithm is an iterative optimization process that uses the point tracks to simultaneously determine the camera trajectory and the cloud of 3D points lying on the surface to be reconstructed [33,34,35]. On the other hand, the Multi-view Stereo method exploits the sparse point cloud and the camera positions given by the SfM step to reconstruct a dense point cloud with the textures of the images [36]. While the 3D structure estimation is obtained by the SfM method, some MVS applications may require a structured and sequential input to reduce errors and improve the accuracy of the 3D reconstruction, especially when the images do not have good quality or lack appropriate illumination [37]. A meshed surface is determined using the dense point cloud and the image colors and textures are finally projected on this surface [35].

This paper describes a method to achieve a 3D anatomical model by exploiting the fusion of IR and visible-light images associated with temperature information. In a comparison of the state-of-the-art, the novelty of this contribution lies in the accurate and automatic segmentation of radiometric arrays based on temperature threshold criteria under certain conditions. The advantage of processing radiometric data instead of a false-color image lies in the fact that the RoI can be delimited in terms of a set of temperature values, while the background can be set to zero. In this sense, it is possible to automatically segment a region with search criteria for a specific temperature range in a one-dimensional array, instead of processing a false-color image for a specific segmentation process. This method can automatically eliminate thermal interferences, detect the region of interest, and enhance the RoI contrast. In essence, the method was designed to automatically obtain merged images in which only the RoI would contain false colors, proportionally to the measured temperature.

The paper structure is as follows: Section 2 describes the instrumental set-up, the acquisition protocol, the radiometric data processing, IR image reconstruction, and the automatic overlapping of the visible-light and IR images, the estimation of the point cloud, and the textured surface and the temperature information association. In Section 3, the results and their analyses are presented, and the discussion and conclusion are provided in Section 4 and Section 5, respectively.

## 2. Materials and Methods

### 2.1. Instrumentation Features

The data were acquired with a Ti32 thermal camera (Fluke, Everett, WA, USA), generally used for industrial applications. The thermal camera is equipped with visible-light and IR sensors. Each capture contains the radiometric information and visible-light images, stored as an IS2 format file. The equipment features are described in Table 4. Additionally, the camera has a handle, with dimensions 27.7 cm × 12.2 cm × 17.0 cm, with an advantage of being transportable to healthcare centers for future studies.

### 2.2. Thermal Calibration

**Figure 1:**
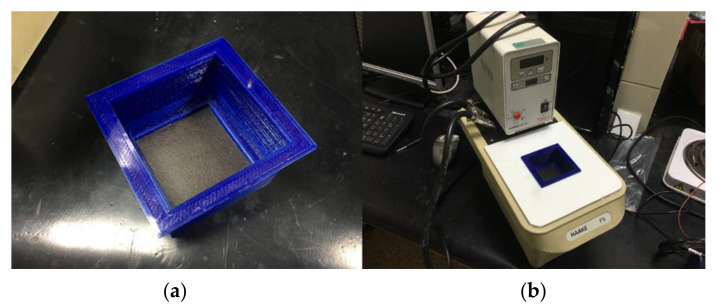
Temperature calibration material. (**a**) Blackbody placed inside a sealed container to isolate the upper surface from the water. (**b**) Thermally controlled water bath with the blackbody placed inside the closed bath container.

To ensure accurate temperature measurements, a calibration was performed with a 10 cm × 10 cm blackbody, which consisted of a matte, black-painted aluminum plate with a smooth texture. The plate was placed inside a sealed container, as shown in Figure 1a. The container with the blackbody was placed inside the thermal bath depicted in Figure 1b.

**Figure 2:**
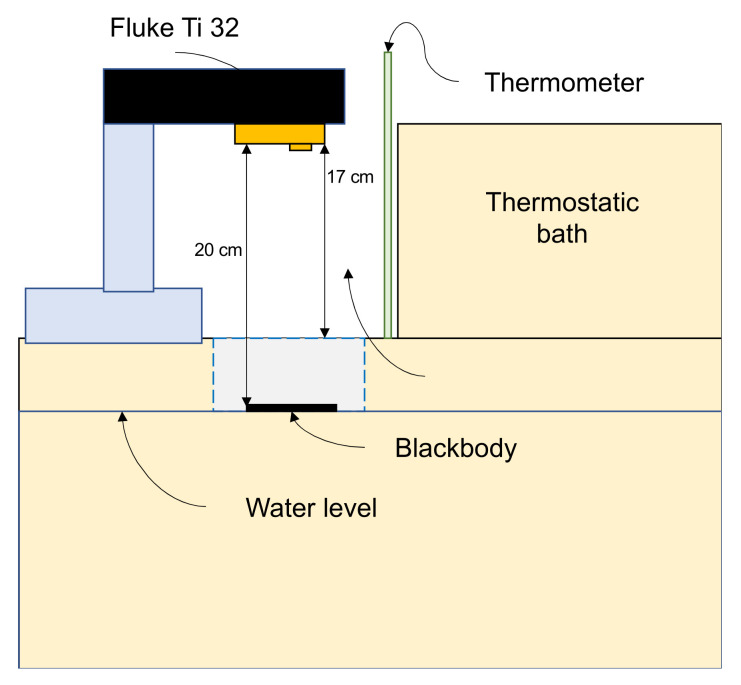
Thermal calibration system.

The thermal camera emissivity was set to 98%, as this value corresponds to the human skin thermal property, which could be considered as a blackbody [38]. Then, the sensor was placed 20 cm away from the blackbody surface and a standard thermometer was located inside the container. The water was heated from 22 to 44 °C in 2 °C steps, with a settling time of 2 min at each step for temperature stabilization. The temperature was measured on the center point of the plate, which is expected to have a homogeneous distribution along the surface. Figure 2 describes the diagram of the calibration system.

### 2.3. Acquisition Protocol and Curvature Effect Correction

The volunteer selection was carried out by a public invitation. The samples required for the purposes this first approach only required volunteers indistinctly with a history of DM. The informed consent was presented to the participants under the guidelines of the World Health Organization. Each volunteer was informed that their health, integrity, and personal data will not be exposed and will be kept confidential during and after the publication of this research. Due to the main objective to obtain a 3D model of the foot, the recruitment conditions neither considered age, gender, or background of DM, nor visible alterations on the foot.

**Figure 3:**
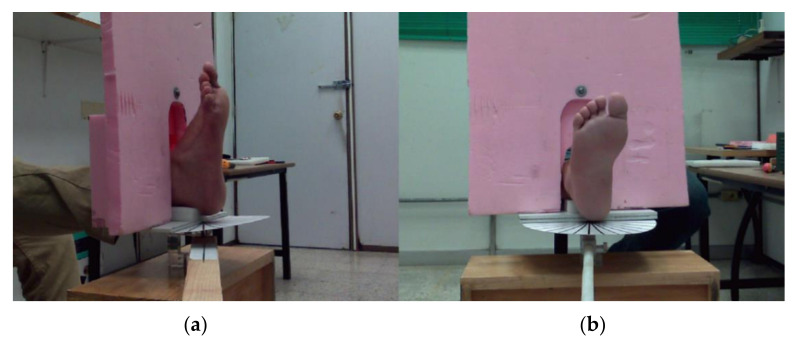
Foot posture during the image acquisition. (**a**) Lateral view of the foot on the resting base with angle and the thermal insulating foam background. (**b**) Frontal view. It is noteworthy that the pink background foam is large enough to isolate the foot from the remaining body of the volunteer so that the IR radiation does not interfere with the captured frame.

Each volunteer was seated with the right lower limb in a straight position and was instructed to remain as steady as possible. The foot rested over a support base with an angle guide and thermal insulating background, as illustrated in Figure 3. The proposed protocol was based on passive thermography, in which the foot was exposed to room temperature for 15 min to generate a thermal equilibrium and avoid drastic temperature changes and the samples were taken in a radial sequence. Sudden temperature fluctuations could cause surface distortions or loss of correlation at the feature-matching stage between two consecutive images.

**Figure 4:**
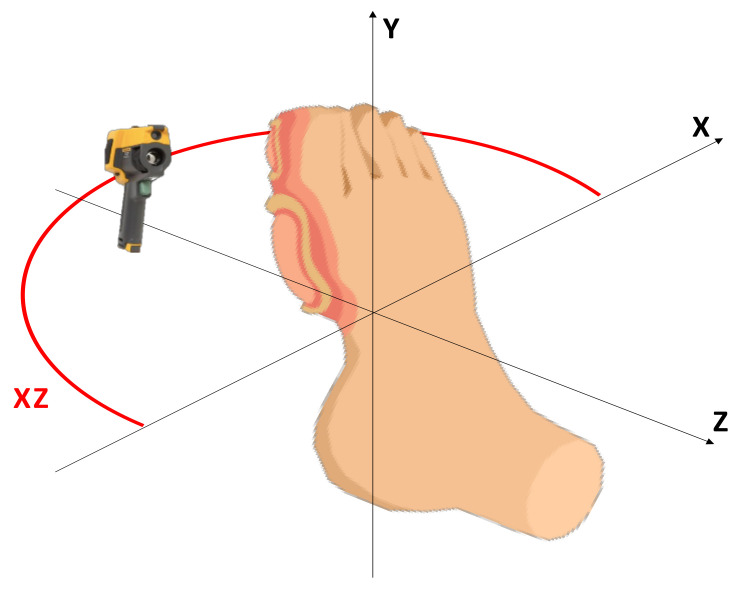
The sequence trajectory in the XZ plane is represented by the red arc. The camera was placed in a vertical position regarding the foot.

The camera was placed at 95 cm from the foot, in a parallel position, and rotated every 12° around the resting chair in order to obtain 15 captures per volunteer, i.e., 15 visible-light frames and 15 radiometric data arrays from left to right viewpoints of the foot. The equipment was stabilized with a tripod to avoid motion artifacts and the time between the acquisition of two consecutive samples was 13 s. The capture timing prevented spatial or thermal blurring. Additionally, with the controlled environmental conditions, airflows are avoided, so that the acquired surfaces do not have drastic temperature variations. The acquisition trajectory is illustrated in Figure 4.

A limitation in temperature acquisition with a curvature trajectory is the susceptibility to angular emissivity despite the uniform temperature distribution along the skin surface. This physical factor impacts the relative temperature recording, inducing errors for each capture at different angles [39]. To deal with such issues, there are models for an estimation of the errors due to the viewpoint angle. The approach proposed by Cheng et al. [40] suggests a linear model related to directional emissivity for the error measurement, as described in Equation (1):

(1) $$\Delta T=\frac{T_{n}-T\left(\theta \right)}{T_{n}-T_{a}}$$

where, ΔT is the temperature error normalized to the range [0,1], $T_{n}$ is the temperature measured at a normal angle (i.e., 90°), $T\left(\theta \right)$ is the temperature obtained at any viewing angle different from the normal angle, and $T_{a}$ is the room temperature (i.e., 20 °C for our study).

**Figure 5:**
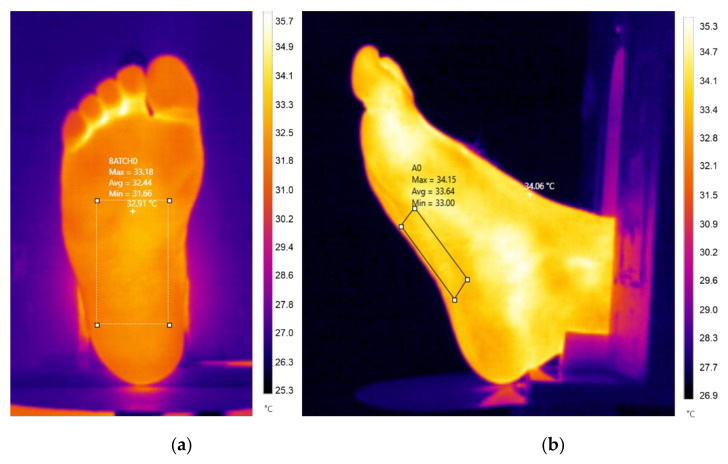
Thermal images taken from different viewpoints. (**a**) Reference sample at a normal angle. (**b**) The last sample at a 90° angle change position. For each sample, the average temperature was retrieved within a marker box.

For each sample, the average temperature was retrieved between the metatarsal and heel zone since they presented a homogeneous distribution, and the area is visible within each capture (see Figure 5). A total of 8 samples were taken for each dataset, from 0° to 90°.

### 2.4. IR Radiometric Data Extraction and Processing

A specific feature of the IRT technique is that the object of interest is warmer or cooler than the background in certain environments [41,42]. In medical thermography, the limb was left to cool with the room temperature in such a way that the temperature was homogenized [43,44]. However, due to the physiological processes of the human body, the limb usually has a higher temperature than the surrounding environment. In addition, medical thermography protocols, in general, suggest maintaining a controlled environment without continuous air flows and heat sources at the scene [15,43].

**Figure 6:**
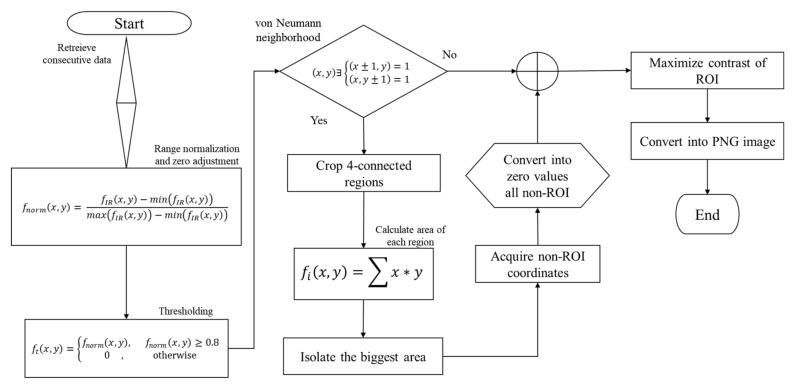
Flowchart of the radiometric data processing to obtain a segmented IR image, without interferences or non-desirable artifacts.

In terms of data processing, this feature enables a high contrast between the RoI and the background, leading to a relatively easy way to segment the RoI. However, the capture may be affected by heat interferences due to several external factors, such as lamps, electronic equipment, surrounding people, thermal shadows, reflections, or even the volunteer body heat. The flowchart presented in Figure 6 summarizes the overall process for extracting and processing the information. The input is a temperature matrix, while the output is an IR false-color image with the segmented RoI in jet colormap scale (blue to red). Each radiometric array was represented as a color-scaled image in which the colors have a direct correlation to the temperature intensity measured in each frame.

Although the thermal camera has a management software (SmartView), an automated processing for our specific purpose such as the segmentation of the RoI is not available.

The data was extracted from each IS2 file with a version of the READIS2 Matlab code, written by Beauducel et al. [45]. The code was modified for this paper, i.e., extract the 14-bit radiometric data interpreted as temperature given in degrees Celsius and exported as text (TXT) format, while the visible-light images were extracted in Portable Network Graphics (PGN) format.

The first step was to perform a normalization of the radiometric data *f_IR_*(*x*, *y*) so that the value *f_norm_*(*x*, *y*) belongs to the [0,1] interval, see Equation (2):

(2) $$f_{norm}\left(x,y\right)=\frac{f_{IR}\left(x,y\right)-\min\left(f_{IR}\left(x,y\right)\right)}{\max\left(f_{IR}\left(x,y\right)\right)-\min\left(f_{IR}\left(x,y\right)\right)}$$

After the normalized data are thresholded, the values below 0.8 are set to 0, whereas the other values remain unchanged, see Equation (3):

(3) $$f_{t}\left(x,y\right)=\left\{\begin{array}{c} f_{norm}\left(x,y\right),\;\;\;\;\;\;\;f_{norm}\left(x,y\right)\geq 0.8\\ 0\;\;\;\;\;,\;\;\;\;\;\;otherwise \end{array}\right.$$

where $f_{t}\left(x,y\right)$ is an array with thresholded values. The values below 0.8 were set to zero, converting them as background components, meanwhile, the values higher than 0.8 were kept as their normalized original value. Equation (3) homogenizes the components of the background, removing several interferences in the background. However, some IR interference may be strong enough to remain present in the background of the thresholded array $f_{t}\left(x,y\right)$. In the binarized image *f_bin_*(*x*, *y*), these artifacts correspond standardly to isolated objects with a small area with respect to the RoI. Equation (4) describes the detection of the regions with enough temperature intensity that remains in $f_{t}\left(x,y\right)$:

(4) $$f_{bin}\left(x,\;y\right)=\left\{\begin{array}{c} \;\;\;\;\;\;\;1,\;\;\;if\;f_{t}\left(x,y\right)>0\\ 0,\;\;\;otherwise \end{array}\right.$$

Such non-desirable objects and the RoI, denoted by *f_obf_*(*x*, *y*), were detected as 4 connected neighborhood regions with a Manhattan distance of r = 1. This step allowed to segment the RoI and the interferences as islands that have a similar temperature. The RoI is always located in the center of the frame with a greater area than the non-desirable objects due to the IR interferences. For this reason, instead of using morphological operators such as erosion and dilation, the largest and central area was delineated to find the sub-image, i.e., the foot. The values of the pixels corresponding to non-RoI objects are simply set to 0. The binarized images are essential for retrieving the coordinates of the RoI, to extract the original values of the thresholded array $f_{t}\left(x,y\right)$ that correspond only to the foot. Afterward, the data of the sub-array from $f_{t}\left(x,y\right)$ was displayed as a false-color image with a Jet colormap range from blue to red. The blue color corresponds to the lowest value (i.e., zero) while the rest of the colors were mapped only on the RoI.

**Figure 7:**
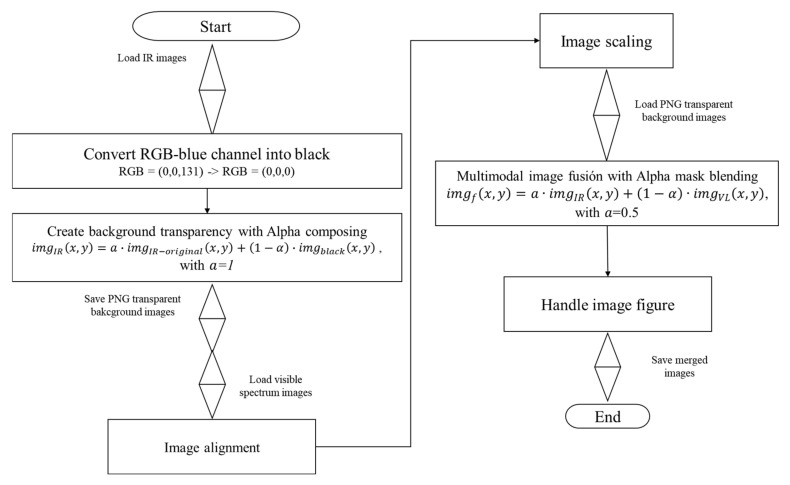
Flowchart of the multimodal co-registration. For each IR image, the blue background was transformed into black to apply the alpha mask transformation. The visible-light images were co-registered with the IR images by alignment, scaling, and merging.

The following process was performed to co-register the IR image onto the visible-light image. Since the thermal camera is equipped with multi-modal sensors, the foot can be captured in a single shot. The main reason for considering the images in visible light is that contrary to the IR images, the visible-light images contain texture and structure information, which contains feature points and descriptors. This information can be determined for a robust and accurate surface reconstruction using an SfM approach, so the estimation of the structure process could use the visible-light images as a reference instead of using only IR images. Figure 7 describes the fusion process between both modalities.

Considering that the background of the IR images is blue color, the component RGB = (0,0,131) was tracked in each pixel to replace the value into black color, i.e., RGB = (0,0,0), since the alpha mask described in Equation (5) will make transparent only with black pixels. Therefore, after performing the alpha mask, the output will be an image with a perfect segmented RoI and transparent background.

(5) $$img_{IR}\left(x,y\right)=a\cdot img_{IR-original}\left(x,y\right)+\left(1-\alpha \right)\cdot img_{black}\left(x,y\right),\;\mathrm{with}\;a=1$$

where $img_{IR}\left(x,y\right)$ is the target image with transparent background, $img_{IR-original}\left(x,y\right)$ is the IR with a black background, $img_{black}\left(x,y\right)$ is the background base color, and *α* is the transparency factor *α* ∈ [0,1].

**Figure 8:**
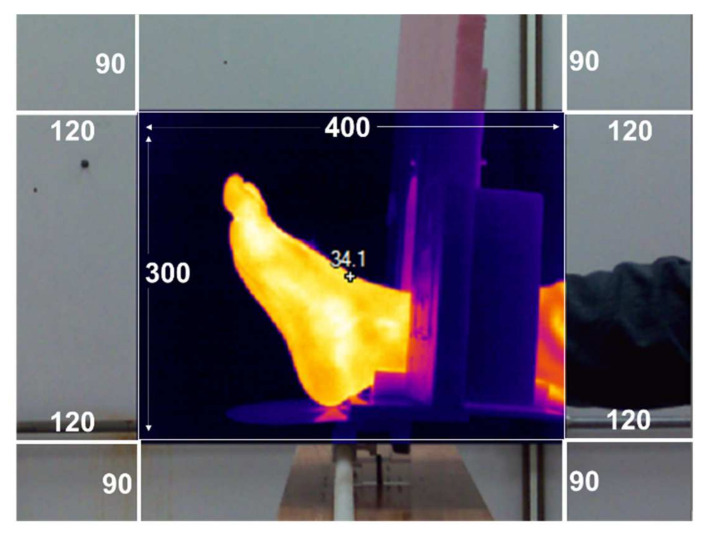
Multimodal superimposed images without RoI segmentation.

Once the RoI was retrieved with a transparent background, the following step was performed to co-register the source image onto the visible-light target image. An advantage of a bifocal system with sensors fixed in the same position is that their scales will be constant. Therefore, the visible-light images were resized 1.25 times to match the resolution of the IR images. Then, the source images were cropped to 400 × 300 since both modalities are aligned inside this spatial region (see Figure 8.)

### 2.5. Estimation and Reconstruction of the 3D Model with SfM and MVS

Once the multimodal images were merged, they were implemented as input for the 3D reconstruction stage. The 3D structure estimation search corresponds on the background of the visible images as a reference to determine the 3D sparse point cloud. Therefore, using only false-color images, it is not possible to obtain a correct correspondence, so the retrieved point cloud will not be accurate or null. The 3D reconstruction process was carried out with the COLMAP software [34] for retrieving the point clouds and camera positions, while the reconstruction of the dense point cloud, the mesh determination, and the surface texturing was carried out with Open-MVS libraries [46]. The surface reconstruction algorithm consists of the following steps:

- Image pre-processing: This step is described in Section 2.4. The false-color images are retrieved after segmenting the foot based on thresholding criteria. The RoI is mounted into the visible-light images by scaling and translation. The output of this step is a set of merged images of both modalities (i.e., IR and visible light).
- SfM: The merged images were converted into the gray level domain by modeling a weighted addition of the R, G, and B components. Then, the sparse 3D point cloud and the camera parameters (i.e., position and orientation) were retrieved in this step. The point cloud is obtained by a cluster of homologous 2D points from the projection of the same point on different viewpoints, which are used for the estimation of the point cloud and camera poses [34].
- Dense reconstruction: This step retrieves depth and maps for all co-registered images to fuse them with the dense point cloud. Then, a dense surface is estimated from the fused point cloud using Poisson surface reconstruction [47].
- Mesh generation: an estimated surface is obtained by triangular facets from the dense cloud, based on the mesh-generation algorithm [46].
- Surface texturing: a sharp and accurate color texture of the images is superimposed on the mesh surface [46].

The 3D reconstruction accuracy of the SfM algorithm used in this contribution was assessed in [35,48] using a phantom with known dimensions and shape. This phantom was covered with paper sheets on which human skin images were printed to simulate in a realistic way textures of a healthy foot and hand epithelium. The 3D surface of this skin textured phantom was reconstructed using the SfM algorithm. For a surface having the size of a foot, the mean distance between real and reconstructed point positions is systematically less than a millimeter. This 3D point reconstruction error is low enough to ensure an accurate shape reconstruction.

An advantage of using COLMAP is the robustness and accuracy for the surface reconstruction, even if the intrinsic camera parameters are unknown [49]. For our application, the exact scale of the surface is not relevant for the 3D foot representation. During the surface construction, the COLMAP software computes intrinsic camera parameters (notably the radial distortion parameters, see the first line in Table 5), the 3D camera motion (using the external camera parameters), and a sparse set of 3D points located on the surface of the objects. The parameter settings for COLMAP software are provided in Table 5.

The sparse 3D point cloud and camera positions were imported in the Open-MVS libraries proposed by Cernea [46]. The dense point cloud is used to construct a mesh using the Poisson surface reconstruction method to retrieve a 3D surface model. The input for this process is the position of the camera and the point cloud obtained with SfM. The MVS method ends with the projection of the image textures onto the meshed surface.

### 2.6. Temperature Association

One of the objectives of this work lies in displaying, in an interactive way, the 3D surface with temperature information. The 3D model provided by the reconstruction stage with MVS has a PYL format (Polygon File Format) with a texture map in PNG format. Thus, MATLAB (MathWorks Inc., Natick, MA, USA) was an appropriate option for displaying the results along a color bar related to the temperature information extracted from the radiometric arrays as a proof of concept.

**Figure 9:**
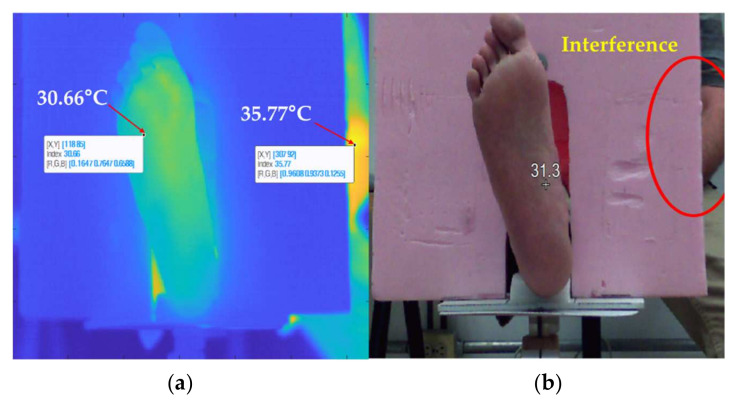
Color contrast mapping according to the warmest spot. (**a**) The hottest spot of the picture frame is located on the upper limb, which decreases the color contrast of the foot image. (**b**) The image was acquired by a wrongly aligned camera since the foam background should completely cover the volunteer’s body.

The first task was to convert the PYL model into an OBJ format so that the triangulated mesh could be imported into MATLAB. The transformation was carried out employing the code proposed by Abayowa [50], which inspects vertices, faces, and texture information from a specified OBJ file. The displayed model can be seen from any viewpoint, i.e., rotations and scale transformations can be performed, displayed along a color sidebar, using which colors are proportional to the temperature values. The mapped colors of each IR image are scaled depending on the hottest point. Figure 9 describes the latter characteristic of the IR thermography technique, which is a disadvantage under uncontrolled background conditions causing thermal interferences. However, due to this property, only the maximum and minimum values may be considered as a reference for adjusting the parameters in the color sidebar. The values are scaled linearly, being red tones for the highest temperature and blue tones for the minimum temperature.

**Figure 10:**
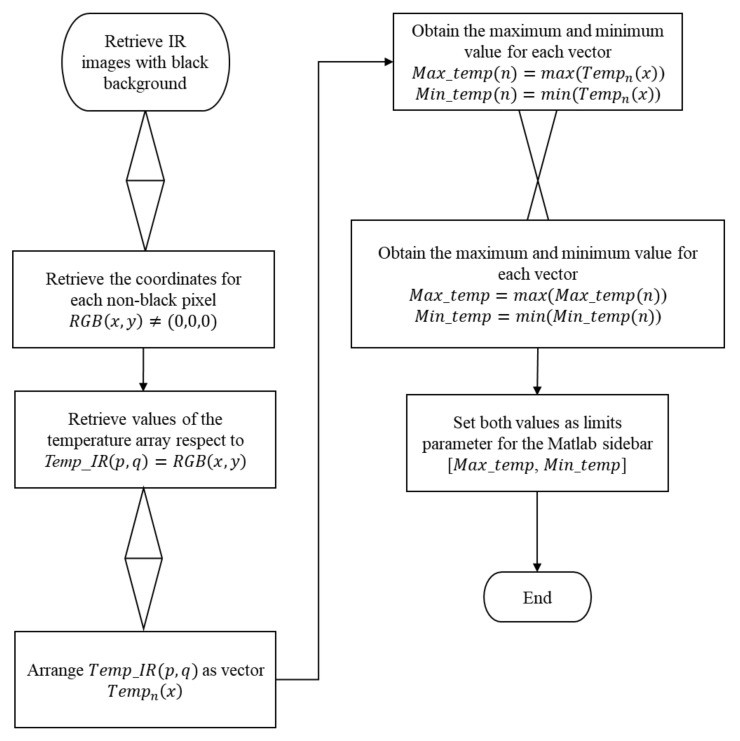
Flowchart for obtaining the limits of the color bar function for the temperature matrix set.

A challenge in associating the temperature with the model was to analyze the radiometric data that only belong to the RoI and discard the background values. Therefore, the structured data were built with non-background values in which every coordinate was retrieved regarding the original temperature matrix. However, each RoI has a different size and temperature values from one array to another (i.e., from one viewpoint to another). An approach for this problem is to retrieve the minimum and maximum limits for each thermal array. Then, the average of the limits was calculated and assigned to the sidebar scale data input and represented by the Jet colormap. Figure 10 summarizes the procedure.

### 2.7. Data for Robustness Testing

**Figure 11:**
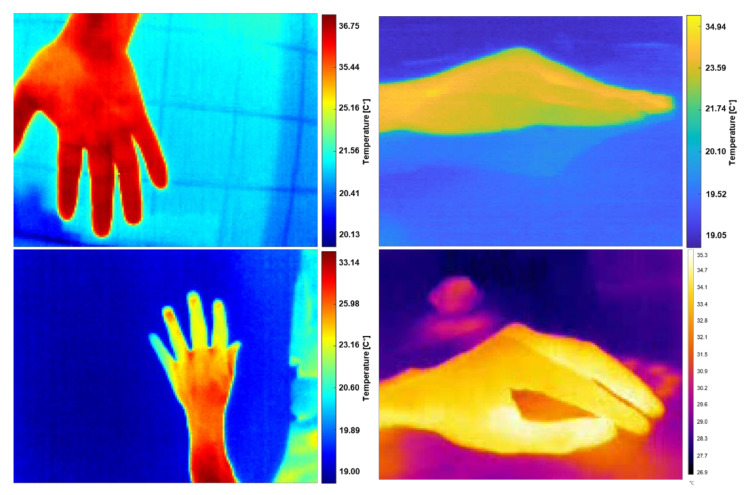
Samples of radiometric information in an uncontrolled environment are represented in false-color images. These samples were used to test the robustness of the automatic segmentation method.

To test the robustness and limitations of the segmentation method, samples of IR radiometric information were obtained in uncontrolled environments on surfaces and backgrounds that would cause thermal interferences. The data were obtained from the hand for the simplicity of having immediate samples in scenarios with possible errors in the capture protocol. The method described in Section 2.4 (see Figure 6) was tested with these samples to remove thermal interferences and segment the RoI maintaining the original scale, and then reconstructed as a false-color image. The data were obtained sequentially by holding the hand motionless while the thermographic equipment was rotated in the normal plane around the hand. Three collections of samples were obtained for different background scenarios and thermal interferences. Figure 11 illustrates some representative samples.

## 3. Results

**Figure 12:**
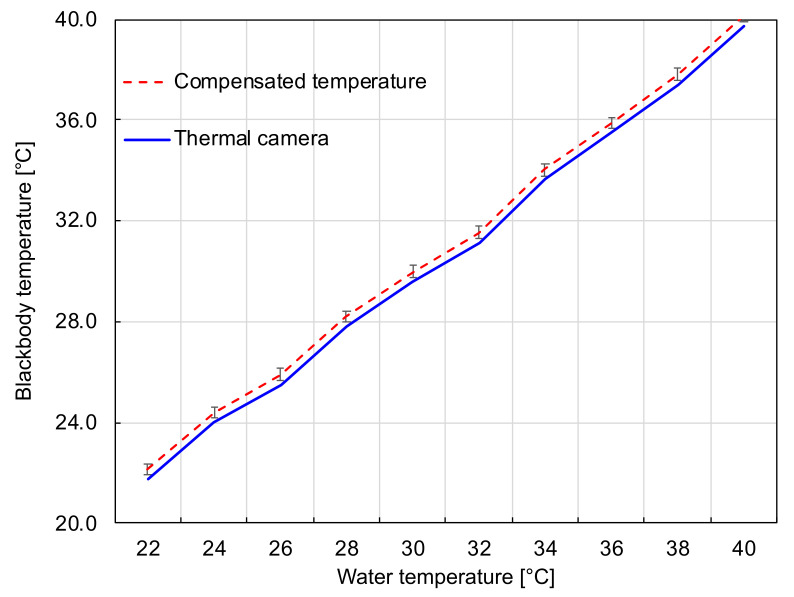
Temperature differences between the measurements of the thermal camera and the standard thermometer. The red dashed line represents the ground truth values and the blue solid curve corresponds to the values measured by the calibrated camera.

A calibration with a known thermal distribution on a blackbody was required to ensure accurate temperature measurements. In this way, the temperature measurement with the camera retrieves values comparable to a direct measurement with a precise thermometer. Figure 12 shows the data recorded by the thermal camera on the center of the plate regarding the water temperature. The average error was 0.4 °C with a standard deviation of 0.2 °C, which is considered acceptable to detect variations on a diabetic foot [51]. The ΔT was adjusted directly on the temperature matrix.

**Figure 13:**
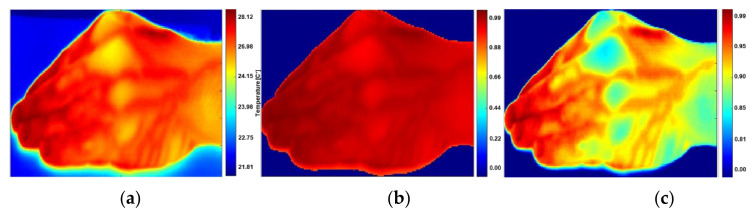
Normalization and segmentation obtained in Section 2.4. (**a**) False-color image with thermal interferences, (**b**) IR image after thresholding step at 0.8. The thresholding leads to a homogeneous image background. (**c**) Segmentation results with color contrast on the RoI, in which the false colors represent the temperature intensities.

Once the ΔT was adjusted, the segmentation method was tested under uncontrolled conditions to prove the robustness of the algorithm. For this purpose, radiometric data with thermal interference and reflections were acquired. Figure 13a shows a raw IR image in which thermal shadows and border blurs can be observed on the fingers and the border of the hand, respectively. Figure 13b is the result after the normalization and thresholding steps described in Section 2.4. In this image, the background is homogeneous and the RoI has low contrast. Figure 13c shows the result after the normalization of the RoI values. Each temperature matrix was visualized as a false-color image to illustrate the results.

**Figure 14:**
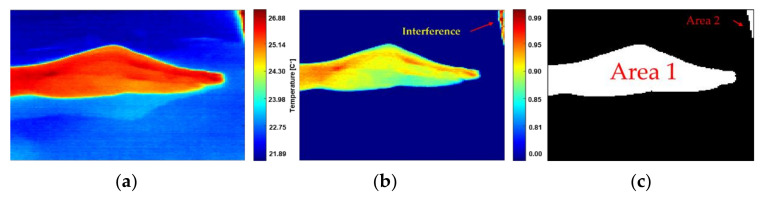
Illustration of the inference treatment. (**a**) Raw IR image with thermal interferences and reflection, (**b**) normalized radiometric data with interference on the corner, (**c**) area 1 and 2, correspond to warm and large areas with ∆T < 2.6%, but only the area 1 should be labeled as RoI.

After the segmentation and contrast stage, there is still a possibility of interferences due to warm and large objects which are (accidentally) in the field of view, which may lead to strong intensity signals into the images. However, the RoI cropping method described in Section 2.4 allows discarding the interference, which is always located at the image borders and is smaller than the RoI (Figure 14). Smaller thermal interferences located at the borders can also be detected and removed from the images, as illustrated in Figure 14b,c.

**Figure 15:**
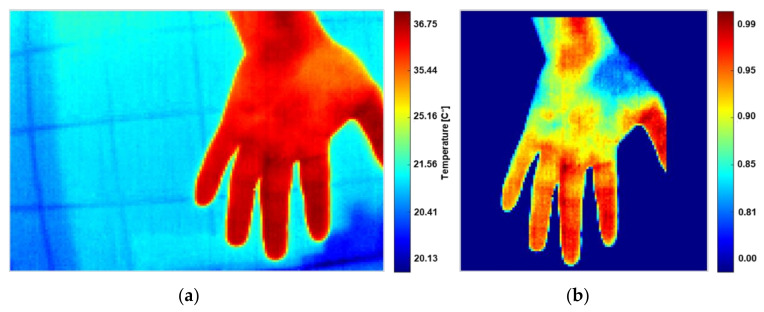
(**a**) Raw IR image, (**b**) segmentation of the RoI, and removal of artifacts.

At this point, IR sub-data provides a RoI including a homogenous background instead of processing a raw IR image, as illustrated in Figure 15. The reconstructed images from the radiometric data are an interpretation of a color distribution related to the temperature intensity measured by the sensors.

Although the proposed method can correctly segment the RoIs, the technique has limitations. The temperatures lying in RoIs correspond to values higher than 0.8 after the normalization, leading to [0,1] intervals, while the remaining temperatures are set to zero. If an interference has a higher temperature of at least 2.6% greater than the RoI, the segmentation method will consider the RoI as a background region. However, if the interferences have a temperature difference under 2.6%, they will be removed by discriminating the larger region in the capture frame. Due to this limitation, it is recommended to either have a thermal insulating background or to avoid thermal artifacts such as light sources, or persons nearby the experimental system. This limitation was illustrated in Figure 9.

**Figure 16:**
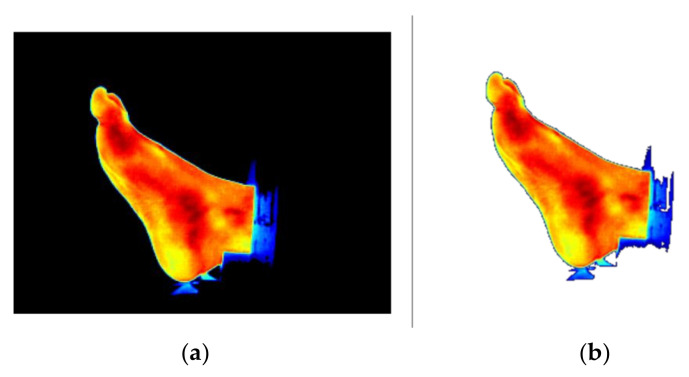
Illustration of the transparency process. (**a**) IR image with a black background and (**b**) IR image with a transparent background.

After the RoI segmentation, the background of the sub-image was transformed into a transparent region. Every pixel with value 131 in the blue channel was transformed into a black pixel (Figure 16a) and then masked with maximum transparency (Figure 16b).

**Figure 17:**
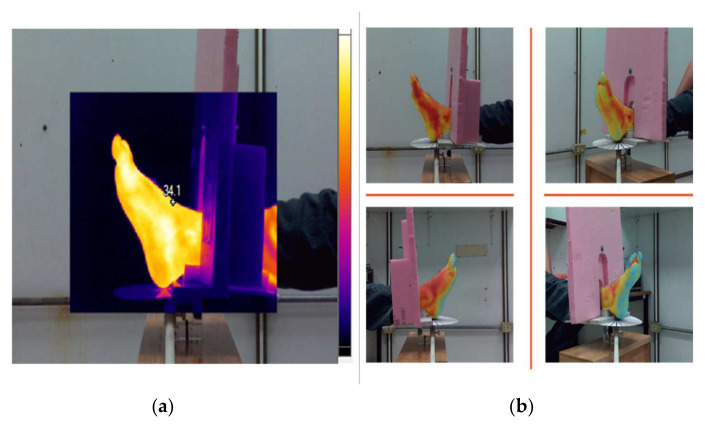
Multimodal image representation: (**a**) SmartView Picture-In-Picture image in which the IR is superimposed on the center of the visible-light image. (**b**) Results of the merged stage, provided by an IR image with a transparent background and the visible-light image as the scene.

As illustrated in Figure 17a, the software of the Ti32 camera has a Picture-In-Picture which superimposes the images of both modalities. Even with this center superimposition where both common fields of view are overlapped, the background of the IR image remains, which is irrelevant for our purposes. Figure 17b shows that, contrary to the Ti32 software (SmartView), the proposed superimposition method allows to observe only the foot in the IR image modality from any viewpoint, while having the standard white light information in the remaining part of the scene.

**Figure 18:**
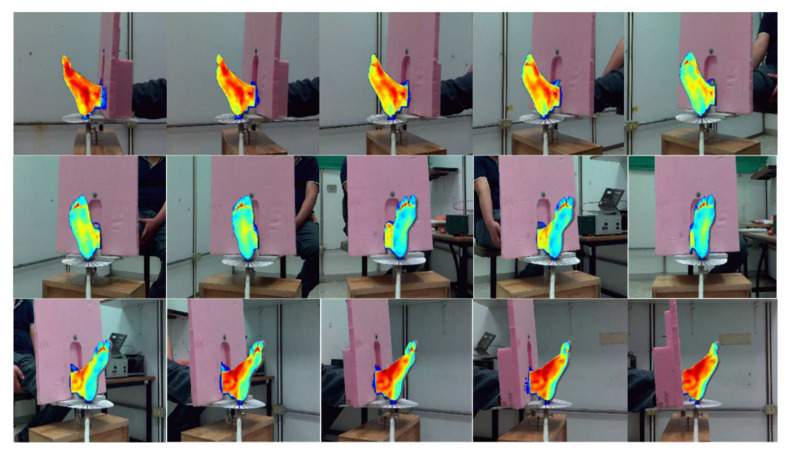
Set of 15 merged image pairs used for determining the 3D point cloud.

Figure 18 shows a representative collection of 15 multimodal merged images, in which the IR-RoI was accurately co-registered for all viewing angles regarding the corresponding region in the visible-light image, without using manual mounting by software or RoI detection by classification.

Although every radiometric data array has a dimension of 320 × 240, the RoI has a different dimension depending on the angle of capture, as noticeable in the first columns of “Image set S1” and “Image set S2” of Table 6. The percentages of these RoI pixels generally represent less than one-third of the original matrix size. Consequently, all 76,800 values are not processed or involved in the superimposition process.

**Figure 19:**
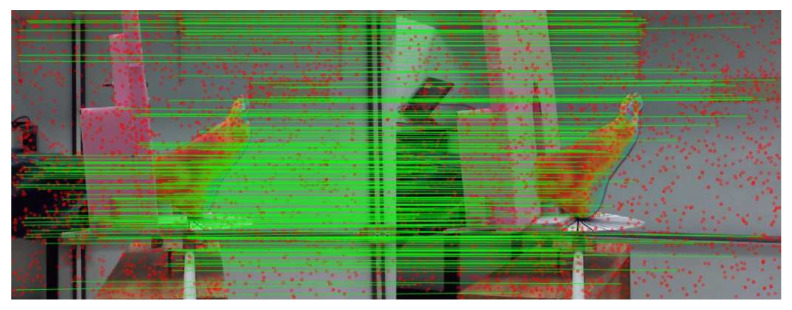
Detected feature points (red dots) and visualization of their correspondence (the green lines represent the link between homologous points). This figure represents 202 matches between images 6 and 7 from set S1, in which most of the key points were dismissed, proving that the quality of the model depends on the accuracy of the matching points’ process.

It is recalled that feature points are detected using the SIFT algorithm and their feature descriptors are used both to find homologous points between image pairs and to track this homologous point along with all images of the set of acquired data. The results have shown (see Figure 19 and Table 7) that for the SfM algorithm, it is not only the correspondences for the IR images that are important but also the matched point pairs are required for the pixels of the visible-light images. Even if only homologous IR points pairs are finally used by COLMAP to reconstruct the sparse point of the foot surface, the determination of visible-light homologous point pairs (spread over the complete image, except in the center) is required for a precise camera displacement determination (external camera parameters) which impacts the 3D points’ reconstruction accuracy.

**Figure 20:**
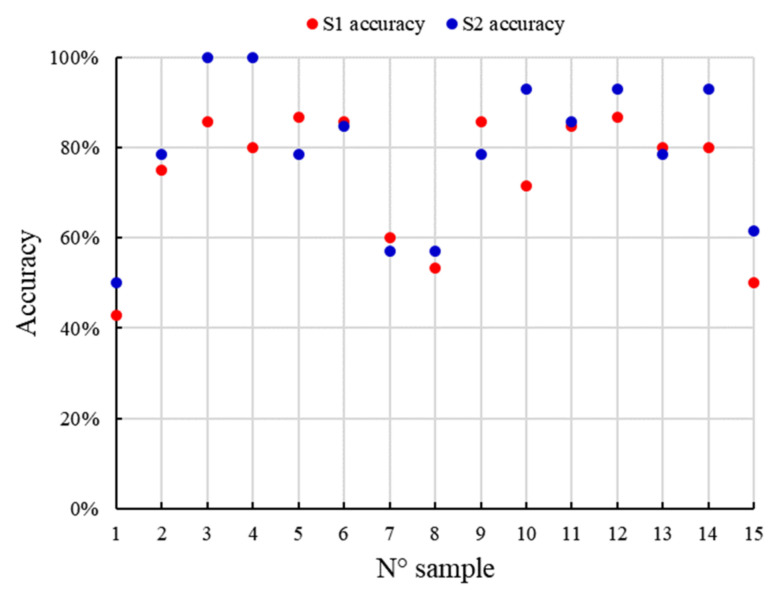
Geometric verification accuracy for each S1 and S2 image set of the foot.

Figure 20 presents the accuracy for the geometric verification stage. The accuracy was determined between the matching step with the SIFT data and the potentially overlapping image pairs for a rotating camera capturing on a planar scene and RANSAC as a robust technique for the estimation of correct correspondences. The accuracy between the number of images in the matching stage and the geometrical verification was 77% ± 2% for both image sets. It is noteworthy that the lateral and central images (i.e., 0°, 90°, 180°) have fewer correspondences.

**Figure 21:**
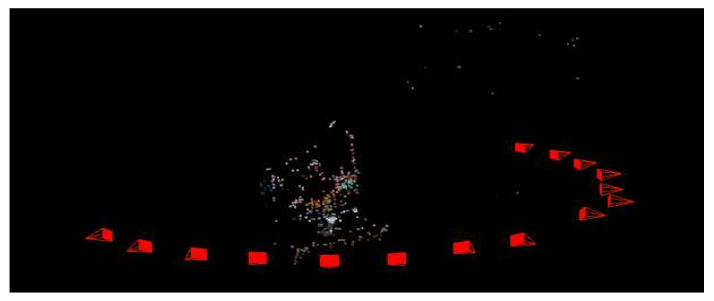
Successive camera poses (the vertex of the red tetrahedron indicates the camera optical center position while the complete tetrahedron represents the camera orientation). This figure also shows the sparse 3D point cloud determined by COLMAP.

Robustly matched and accurately homologous point pairs of the visible-light images were not only relevant for the sparse point cloud determination. Indeed, the camera positions also have to be accurately known for the dense point cloud estimation and 3D reconstruction with MVS. Figure 21 shows the positions of the cameras which rotate around the plane. Table 8 shows the statistics of the surface reconstruction performed by COLMAP.

After the 3D point cloud was obtained, the model was exported from the COLMAP software as a file with an NVM format to the Open-VMS software, to reconstruct the dense cloud. The point cloud is used to determine a meshed surface’s reconstruction, refining, and texturing. At the end of the process, a PLY format model is obtained along with its associated texture in PNG format. The computing time of this complete processing chain was:

Image set S1: 2.47 min
Image set S2: 3.12 min

Once the 3D reconstruction of the foot was achieved, the temperature error due to the angular trajectory was compensated. Table 9 shows the errors in percentage concerning the reference sample at a normal angle. The average temperature recorded between the metatarsal zone and the heel increased as the angle increased. For both samples, the error was approximately 10% near 90°.

The error calculated for each angle was compensated in the corresponding thermal map so that each array had the most accurate temperature, as if it had been measured at a normal angle. After compensation, the maximal and minimal temperatures for each set of images were determined. The results of the standard deviation of these maximums and minimums suggest that this proposal could be an adequate strategy to associate the temperature intensity with a color scale since the spread regarding the temperature average is less than 1 °C. Table 10 shows the average temperature calculated for image sets S1 and S2 using their radiometric data arrays.

**Figure 22:**
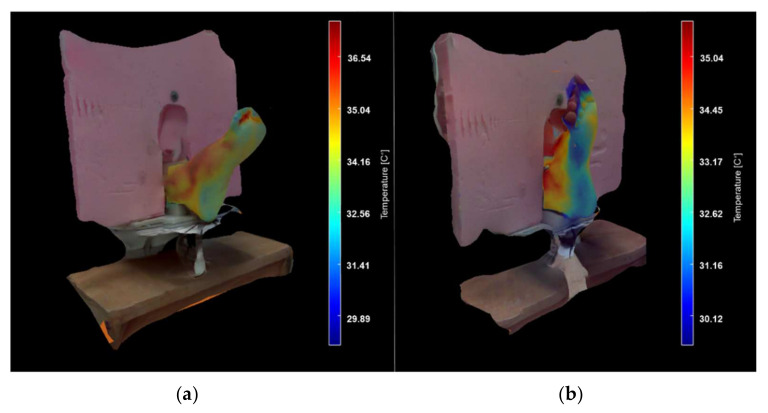
3D models of the foot in a thermal surface and a visible-light environment. (**a**) Volunteer 1 and (**b**) Volunteer 2.

Figure 22 shows the surfaces of both volunteers’ feet, displayed along with the background foam and the support on which the foot was placed. A color bar is displayed with temperature data that associate each color with the surface temperature on the right side of each model. The 3D model displays only temperatures for the foot as wrapping textures, providing a quick inspection of the anatomical and physiological state of the foot under study.

## 4. Discussion

The results prove the feasibility and the relevance of radiometric data extraction and processing, to retrieve a segmented IR image merged in a textured visible-light scene. An IR radiometric processing method can eliminate thermal interference in the background using thresholds as segmentation criteria before interpreting thermal maps as images. This approach leads to automate the process regardless of the targeted RoI, under certain limitations: a controlled environment and the exclusion of heat sources in which the ΔT < 2.6% compared to the RoI. Under other conditions, the segmentation with thermal arrays would be inadequate. However, medical thermography protocols suggest sampling in a controlled environment to avoid interferences. Our work proves that when these recommendations are followed, the proposed method is accurate.

An advantage was that the thermal equipment provides a solution for the parallax problem, so no additional thermal/spatial calibration was required. Additionally, the dimensions would provide portability for future clinical validation so the device can be transported to health centers for future clinic validation. Only a thermal calibration on a body with known thermal distribution was carried out against a standard thermometer, in which ∆T = 0.4 °C was corrected. In addition, the ∆T error due to the angular emissivity factor was compensated (error ≈ 10% near to 90°), a problem that is usually encountered in 3D thermography studies. Without an angle compensation, a distribution error of 4.74 °C was found on average for both sets.

It is important to point out that the RoI detection was neither based on a specific segmentation of the foot nor on the implementation of a neural network. Thus, one of the advantages of this method is that it can be adaptable to other applications in terms of RoI segmentation with radiometric data, such as portable IR thermography devices and prototypes based on embedded systems. However, a registration and calibration method will be required for both sensors. For instance, this study was carried out with thermographic equipment designed for general/industrial purposes, which dimensions and handling could allow carrying into research laboratories or health centers for future studies. For all these reasons, it is expected that the proposed method can be applied to any thermographic equipment acquiring radiometric information.

Regarding the surface reconstruction, the COLMAP algorithm requires an accurate feature detection and matching step which cannot be warranted when only IR images including few textures are used. As a consequence, the visible-light images were essential as additional data to enable the SfM method to construct surfaces robustly and accurately. The average accuracy was 77% ± 2% for both image sets, calculated after the RANSAC correction regard the SIFT matching. The accuracy of a 3D surface depends on the quality of the images, rather than the number of key points, as the 3D model for the S2 set has proven. The set S1 had 42% fewer key points than S2, but the reconstruction was visually more accurate (see Table 8). A possible solution may be improving the visible-light sensor by including modern cameras with a higher resolution and smaller pixel size. However, the IR radiometric arrays were fundamental to determine IR images which were exploited as a “wrapping texture” approach, since they only cover the surface of the model in which the color distribution is directly related to the temperature intensity. Since the model is supplied with a temperature scale, it is pertinent to accurately assign the color distribution proportionally to the temperature intensity of the RoI. The temperature was compensated concerning the angle of capture since this is a factor in thermography due to angular emissivity. The ∆T according to the reference sample was suggested to be a linear problem, so the temperature matrix accuracy was compensated by using Equation (1). The error adjustment did not influence the color distribution in the RoI but did influence the scale along with the 3D model.

## 5. Conclusions

IR medical thermography is recognized as an appropriate technique to detect temperature changes and to assess the evolution of diseases and complications, such as the diabetic foot. In this paper, a method for the 3D surface reconstruction of the foot displayed with a temperature scale was presented, aimed at diabetic foot treatment and diagnosis. The contribution of this work, concerning the most common techniques described in the state-of-the-art, is the automatic processing of IR radiometric without relaying to manual manipulation. Automatic processing has the advantage of treating all data and image arrays under the same segmentation criteria, which also avoids individual manipulation that can be more time-consuming and is often subject to user interpretation.

The segmentation method is accurate under certain conditions: the RoI should be warmer than the background to threshold values higher than 0.8 in a range-normalized array [0 to 1], and if the background thermal interferences are ∆T < 2.6%, regard the RoI (otherwise, the RoI would be transformed into a background component). Due to this limitation, it is recommended to either have a thermal insulating background or to avoid thermal artifacts such as light sources; moreover, the personnel themselves could produce thermal artifacts.

In essence, our method for automatic segmentation and registration was designed to obtain multimodal merged images in which only the RoI would contain false colors, proportionally to the measured temperature. In such a way, the 3D structure estimation algorithm would use the background components (which are visible light) as a reference to determine the 3D sparse point cloud, and the RoI would be exploited as a “wrapping texture” approach. Otherwise, using only false-color images, it is not possible to obtain a correct correspondence, so the retrieved point cloud is not accurate or is sometimes null. The quantity of matched images was 77% ± 2% for multimodal images after the geometric verification method.

Additionally, the ∆T error due to the angular emissivity factor was compensated and the temperature was associated with the reconstructed 3D surface to facilitate the inspection of the physiological state of the foot, and provides additional diagnosis criteria.

## Figures and Tables

**Table 1:** State-of-the-art of medical imaging for DM complication studies on lower limb and foot.

Author	Year	Medical Imaging Technique	Application
Short and Zgonis [7]	2017	Tomography and MRI	Diabetic Charcot neuroarthropathy
Ramanujam et al. [8]	2018	Radiography and MRI	Diabetic Foot Osteomyelitis and Partial Foot Amputations
Toledo Peral et al. [9]	2018	Digital image processing	Skin Macules Characterization
Goyal et al. [10]	2020	Image-based machine learning algorithms	Recognition of ischemia and infection
Maldonado et al. [11]	2020	Image-based machine learning algorithms and thermography	Diabetic foot necrosis detection
Bayareh Mancilla et al. [12]	2021	Radiometry data and digital image processing	Detection of regions with non-homogeneous temperatures

**Table 2:** Overview of recent studies regarding the registration between visible and thermal images.

Author	Year	Method	Application
Liu [24]	2017	Convolutional neural networks	Multi-modal medical image fusion aims
El-Hoseny et al. [25]	2019	Non-sub-Sampled Shearlet Transform and Modified Central Force Optimization	Object detection and medical diagnosis
González-Pérez et al. [20]	2021	Geometric Optical Translation, Homography, Iterative Closest Point, and Affine transform with Gradient Descent	Diabetic foot monitoring

**Table 3:** Overview of the 3D reconstruction techniques based on visible-light images and/or IR images. Although the acquisition techniques are different, the objective is common for all the reported papers for a 3D structure estimation.

Author	Year	Method	Optical Technique	Data/Image Processing	3D Structure Estimation	Acquisition	Application
Souza et al. [27]	2015	Structured light	Active	Particle Swarm Optimization	Structure from motion	Sequential frames	General purposes
Chernov et al. [28]	2017	Stereoscopy	Active	Correlation between depth and IR images	Structure from motion	Non-sequential multi-frame	Breast reconstruction
van Doremalen et al. [29]	2019	Stereoscopy	Passive	Projective transformation	Not reported	Non-sequential multi-frame	Diabetic Foot study
de Queiroz Júnior and de Lima [30]	2020	Stereoscopy	Passive	Manual tracing of the profile curve	Not reported	Sequential frames	Breast pathologies study

**Table 4:** Fluke Ti32 Commercial Thermal Imager characteristics.

Characteristics	Range	Units
Visible-light sensor resolution	480 × 640	Pixel
Infrared sensor resolution	240 × 320	Pixel
Temperature Range	−20 to +600	°C
Thermal Sensitivity	≤50	mK
Infrared Spectral Band	8–14	µm
Minimal focus distance	46	cm
Refresh rate	60	Hz

**Table 5:** COLMAP parameters for feature detection, matching, and point cloud estimation.

Parameter	Value/Option
Camera model	Simple Radial
Guided Matching	Activated
Edge threshold	50
Peak threshold	0.00067

**Table 6:** RoI elements of the images collection S1 and S2. It is noticed that not all regions of interest have a constant number of elements.

Image N°	Image Set S1 (Volunteer 1 Foot)		Image Set S2 (Volunteer 2 Foot)	
	RoI Elements	RoI %	RoI Elements	RoI %
1	14,361	18.7	18,084	23.55
2	16,020	20.86	17,175	22.36
3	15,681	20.42	15,952	20.77
4	15,677	20.41	17,981	23.41
5	13,567	17.67	16,850	21.94
6	12,740	16.59	15,943	20.76
7	11,770	15.33	14,718	19.16
8	13,004	14.32	14,442	18.97
9	16,069	20.92	13,311	17.33
10	12,100	15.76	15,268	19.88
11	12,341	16.07	16,005	20.84
12	13,173	17.15	16,337	21.27
13	14,476	18.85	14,632	19.05
14	14,079	18.33	12,496	16.27
15	13,641	17.76	10,298	13.41

**Table 7:** Detected and matched features in the two consecutive multimodal images.

Modality	Image Set S1 (Volunteer 1 Foot)	Image Set S2 (Volunteer 2 Foot)
	Detected Features	Detected Features
IR	307,404	99,887
Visible light	465,777	255,421
IR + Visible light	440,978	243,440

**Table 8:** Statistics data retrieved by COLMAP.

Statistics	Image Set S1 (Volunteer 1 Foot)	Image Set S2 (Volunteer 2 Foot)
Cameras	15	15
Images	15	15
Registered images	15	15
Points	907	1573
Observations	2731	4649
Mean track length	3.01103	2.9555
Mean observations per image	182.067	309.933
Mean reprojection error	0.916495	0.955175

**Table 9:** ∆T regarding the angle of measurement. The temperature was compensated in the original thermal arrays with the calculated delta.

Angle of Acquisition (°)	Image Set S1 (Volunteer Foot 1)		Image Set S2 (Volunteer Foot 2)	
	Average Temperature (°C)	∆T (%)	Average Temperature (°C)	∆T (%)
0	32.44	0.00	31.01	0.00
12	32.84	3.22	31.19	1.63
24	32.88	3.54	31.28	2.45
36	33.16	5.79	31.63	5.63
48	33.24	6.43	31.83	7.45
60	33.31	6.99	31.89	7.99
72	33.24	6.43	32.02	9.17
84	33.64	9.65	32.24	11.17

**Table 10:** Average maximum and minimum temperatures for image sets S1 and S2. The temperature standard deviations were not higher than one degree, which can be acceptable to be considered as stable information concerning the passive thermography technique.

Image Set	Average Maximum Temperature (°C)	Standard Deviation (°C)	Average Minimum Temperature (°C)	Standard Deviation (°C)
S1	36.54	0.24	29.89	0.67
S2	35.04	0.56	30.12	0.78
